# A Novel Dimeric Short Peptide Derived from α-Defensin-Related Rattusin with Improved Antimicrobial and DNA-Binding Activities

**DOI:** 10.3390/biom14060659

**Published:** 2024-06-05

**Authors:** Gwansik Park, Hyosuk Yun, Hye Jung Min, Chul Won Lee

**Affiliations:** 1 Department of Chemistry, Chonnam National University, Gwangju 61186, Republic of Korea; lineageyapo@naver.com (G.P.); 5300747yun@hanmail.net (H.Y.); 2 Department of Cosmetic Science, Gwangju Women’s University, Gwangju 62396, Republic of Korea

**Keywords:** antimicrobial peptide, rattusin, antibiotics, DNA binding, cytotoxicity

## Abstract

Rattusin, an α-defensin-related antimicrobial peptide isolated from the small intestine of rats, has been previously characterized through NMR spectroscopy to elucidate its three-dimensional structure, revealing a C2 homodimeric scaffold stabilized by five disulfide bonds. This study aimed to identify the functional region of rattusin by designing and synthesizing various short analogs, subsequently leading to the development of novel peptide-based antibiotics. The analogs, designated as F1, F2, F3, and F4, were constructed based on the three-dimensional configuration of rattusin, among which F2 is the shortest peptide and exhibited superior antimicrobial efficacy compared to the wild-type peptide. The central cysteine residue of F2 prompted an investigation into its potential to form a dimer at neutral pH, which is critical for its antimicrobial function. This activity was abolished upon the substitution of the cysteine residue with serine, indicating the necessity of dimerization for antimicrobial action. Further, we synthesized β-hairpin-like analogs, both parallel and antiparallel, based on the dimeric structure of F2, which maintained comparable antimicrobial potency. In contrast to rattusin, which acts by disrupting bacterial membranes, the F2 dimer binds directly to DNA, as evidenced by fluorescence assays and DNA retardation experiments. Importantly, F2 exhibited negligible cytotoxicity up to 515 μg/mL, assessed via hemolysis and MTT assays, underscoring its potential as a lead compound for novel peptide-based antibiotic development.

## 1. Introduction

The discovery of antibiotics marked a revolution in medical treatment, offering a means to effectively combat bacterial infections through various mechanisms, such as the inhibition of cell wall synthesis by penicillin and vancomycin [1,2], DNA synthesis by rifampicin, and protein synthesis by streptomycin [3,4,5]. These antimicrobial agents have saved countless lives and are critical to modern medicine. However, the widespread and often indiscriminate use of antibiotics has led to an unintended consequence: the emergence and proliferation of antibiotic-resistant bacteria. These bacteria have evolved through mechanisms such as the modification of target molecules, the enzymatic degradation or alteration of antibiotics, and changes in membrane permeability to evade the effects of antibiotics. The rise of antibiotic-resistant strains, such as vancomycin-resistant *Enterococcus* (VRE) [6,7], methicillin-resistant *Staphylococcus aureus* (MRSA) [8,9], and multidrug-resistant *Acinetobacter baumannii* (MRAB) [10,11], represents a growing public health crisis, with infections from resistant strains leading to increased morbidity and mortality rates worldwide.

The escalating challenge of antibiotic resistance has spurred a global effort to discover new antimicrobial strategies that can circumvent these resistance mechanisms. In this context, antimicrobial peptides (AMPs) have emerged as a particularly promising avenue of research [12,13]. AMPs, a diverse class of molecules integral to the innate immune systems of all life forms, exhibit broad-spectrum activity against bacteria, viruses, fungi, and even cancer cells [14,15,16]. Their mode of action, typically involving the disruption of microbial membranes through electrostatic interactions, differs fundamentally from that of traditional antibiotics, thus offering a potential solution to the problem of resistance. Moreover, AMPs are less likely to induce resistance due to their mechanism of action and the high cost to the organism of altering membrane composition [17,18,19].

Recent advances in genomics, proteomics, and computational biology have enabled the identification and characterization of AMPs from a wide range of organisms, from humans to plants and insects [20,21,22]. This has led to the development of novel AMPs through bioengineering and synthetic chemistry, aimed at enhancing their stability, efficacy, and specificity while reducing toxicity toward human cells [23,24]. The design of shorter peptides by identifying and optimizing the active regions of naturally occurring AMPs has become a focal point of this research. Shorter peptides not only offer practical advantages in terms of synthesis and cost but also often exhibit improved antimicrobial activity and reduced immunogenicity [25,26,27].

Rattusin, an α-defensin-related antimicrobial peptide isolated from the rat small intestine, stands at the forefront of this research [28]. The defensins, to which rattusin is related, represent a well-studied class of AMPs noted for their potent antimicrobial activity and role in mammalian innate immunity. The unique structure of rattusin, a C2 homodimeric scaffold formed by five disulfide bonds, contributes to its antimicrobial properties, which include activity against a broad spectrum of pathogens, such as Gram-negative and Gram-positive bacteria, and notably, strains resistant to conventional antibiotics. Previous studies have elucidated the three-dimensional structure of rattusin via NMR spectroscopy, providing a blueprint for the design of analogs with potentially enhanced antimicrobial properties [29,30].

Building on these foundations, the current study seeks to explore the functional regions of rattusin by employing a strategy of sequence dissection and analog design. By focusing on the hairpin loop region, we have identified a peptide fragment that not only demonstrates enhanced antimicrobial efficacy compared to the wild-type peptide but also exhibits significantly lower cytotoxicity toward mammalian cells. These findings highlight the potential of rattusin derivatives as a new class of peptide-based antibiotics, offering hope in the fight against antibiotic-resistant bacterial infections.

## 2. Materials and Methods

### 2.1. Materials and Reagents

The materials used included Rink amide 4-methylbenzhydrylamine (MBHA) resin, 9-Fluorenylmethoxycarbonyl (Fmoc) amino acids and amide resin (GL Biochem, Shanghai, China), 1-hydroxybenzotriazole (HOBt), Diisopropylcarbodiimide (DIC), Trifluoroacetic acid (TFA, Sigma Aldrich, St. Louis, MO, USA), HPLC grade Acetonitrile (ACN, Merck, Darmstadt, Germany), 1,2-Ethanedithiol (EDT), Thioanisole, Dichloromethane (DCM, DAEJUNG, Siheung-si, Republic of Korea), N,N-dimethylformamide (DMF, DAEJUNG, Siheung-si, Republic of Korea), Sodium phosphate (NaH_2_PO_4_, Na_2_HPO_4_), Peptone, NaCl (JUNSEI, 4-4-16 Nihonbashi-honcho, Chuo-ku, Tokyo, Japan), Phosphate buffer saline (PBS), N-(2-Hydroxyethyl)piperazine-N′-(2-ethanesulfonic acid) (HEPES), *o*-nitrophenyl-β-galactoside (ONPG), Dimethyl sulfoxide (DMSO), 3-(4,5-dimethlythiazol-2-yl)-2,5-diphenyltetrazolium bromide (MTT Sigma Aldrich, St. Louis, MO, USA), Dulbecco’s modified eagle medium (DMEM, Gibco, Waltham, MA, USA), Fetal bovine serum (FBS, Capricorn, Shanghai, China), 2,2,2-trifluoroethanol (TFE), 3,3′-Dipropylthiadicarbocyanine iodide (DiSC_3_-(5)), and Fluorescein isothiocyanate (FITC, Sigma Aldrich, St. Louis, MO, USA).

### 2.2. Peptide Synthesis

Rattusin analogs were synthesized using the solid-phase peptide synthesis (SPPS) method. The Fmoc-Rink Amide resin capacity was approximately 0.6–0.7 mmol/g, and the synthesis was conducted on a 0.1 mmol scale. A 20% solution of piperidine in N,N-dimethylformamide (DMF) served as the deprotecting agent to remove the Fmoc protecting group, while dichloromethane (DCM) and DMF were employed for resin washing steps. The amino acid coupling reaction was facilitated by 1-hydroxybenzotriazole (HOBt) and diisopropylcarbodiimide (DIC), each used at two equivalents (eq), and the reaction was allowed to proceed for 2 to 3 h. Following peptide synthesis, cleavage of the peptide from the resin and deprotection of sidechain protecting groups were achieved using a cleavage cocktail of trifluoroacetic acid (TFA), water, thioanisole, and 1,2-ethanedithiol (EDT) in a volume/volume ratio of 87.5:5:5:2.5 at room temperature. The crude synthetic peptide was subsequently lyophilized and stored at −20 °C until purification.

### 2.3. HPLC and LC-MS Analysis

The synthesized peptides were purified by reversed-phase high-performance liquid chromatography (RP-HPLC, Shimadzu, Kyoto, Japan) using a Shim-pack C18 column (20 mm × 250 mm, 5 μm particle size) with detection at 230 nm using a UV detector. The mobile phases consisted of 100% H_2_O with 0.05% trifluoroacetic acid (TFA) as solvent A and 100% acetonitrile (ACN) with 0.05% TFA as solvent B. The molecular weight and purity of the purified peptides were determined using liquid chromatography–mass spectrometry (LC-MS, API2000, AB SCIEX, Framingham, MA, USA).

### 2.4. Disulfide Bond Formation

The rattusin fragments F2, F4, F2-AH, and F2-PH contain one or two cysteine residues that can form a disulfide bond. The F2 peptide was incubated in 50 mM sodium phosphate buffer (pH 7) for 24 h at 4 °C. Similarly, the F2-AH and F2-PH peptides were refolded by air oxidation to form intramolecular disulfide bonds under the same conditions. The reaction progress was monitored using liquid chromatography–mass spectrometry (LC-MS) and quenched by the addition of trifluoroacetic acid (TFA).

### 2.5. Antimicrobial Activity

The minimal inhibitory concentrations (MICs) of the peptides were determined following the broth microdilution method recommended by the Clinical and Laboratory Standards Institute (CLSI). Briefly, bacteria in their mid-logarithmic growth phase were diluted in Mueller–Hinton broth (MHB) (Difco, Franklin Lakes, NJ, USA) and aliquoted into a 96-well microtiter plate at a density of 4 × 10^6^ CFU/well. The assay included both Gram-negative bacteria (*Escherichia coli*, *Salmonella typhimurium*, *and Pseudomonas aeruginosa*) and Gram-positive bacteria (*Bacillus subtilis*, *Staphylococcus aureus*, *and Staphylococcus epidermidis*), as well as methicillin-resistant *Staphylococcus aureus* strains (MRSA: CCARM 3089, CCARM 3090, CCARM 3095). The samples were subjected to a two-fold serial dilution before being added to the wells, and the plate was incubated at 37 °C for 24 h. The MIC was defined as the lowest peptide concentration that prevented visible bacterial growth. All experiments were conducted in triplicate and included appropriate growth and sterility controls.

### 2.6. Membrane Depolarization Assay

The cytoplasmic membrane depolarization activity of the peptides was assessed using the membrane-potential-sensitive dye DiSC3-(5), following methodologies previously described in the literature. *Staphylococcus aureus* was cultured in Luria–Bertani (LB) broth at 37 °C until reaching an optical density (O.D.) at 600 nm of 0.3–0.4, indicative of mid-logarithmic phase growth. The cells were then washed with a buffer containing 20 mM glucose and 5 mM HEPES, and the cell suspension was diluted to an O.D. 600 of 0.05 in the same buffer. Potassium chloride (KCl) was added to achieve a final concentration of 100 mM to equilibrate potassium ion levels. The concentrations used for each peptide in our membrane depolarization assays were based on their respective MICs: 32 µM for F2, 4 µM for Melittin, and 16 µM for Buferin-2.

Fluorescence was monitored using an F-4500 FL fluorescence spectrophotometer (Shimadzu, Kyoto, Japan) with excitation and emission wavelengths set at 622 nm and 670 nm, respectively. Melittin, known for its membrane-disrupting properties, served as a positive control, while buforin-2, which translocates across membranes without causing depolarization, acted as a negative control. Both melittin and buforin-2 were tested at concentrations twice their minimal inhibitory concentration (MIC) levels.

### 2.7. SYTOX Green Uptake Assay

The membrane permeabilization activity of the peptides was determined using the SYTOX Green uptake assay, as described previously. *S. aureus* was cultured in LB broth at 37 °C until it reached an optical density (O.D.) at 600 nm of 0.4, indicative of the mid-logarithmic phase. The cells were then washed and diluted to an O.D. 600 of 0.05 using 1× PBS buffer. The dye (SYTOX Green nucleic acid stain) was added to the bacterial suspension at a concentration of 1 mM, and the mixture was incubated at 4 °C for 18 h with agitation in the dark. The SYTOX Green-treated bacteria were placed in a quartz cell, where they were simultaneously exposed to the positive control melittin (2× MIC), the negative control buforin-2 (2× MIC), and the F2 dimer (2× MIC). An F-4500 FL fluorescence spectrophotometer (Shimadzu, Kyoto, Japan) was used to monitor fluorescence (excitation 485 nm, emission 520 nm).

### 2.8. ONPG Hydrolysis Assay

The internal membrane permeability was assessed using the ONPG hydrolysis assay, as described previously. *E. coli* ML-35p, which constitutively expresses cytoplasmic β-galactosidase and periplasmic β-lactamase, is resistant to ampicillin and deficient in lactose permease. *E. coli* ML-35 was incubated in LB broth containing ampicillin at 37 °C until the optical density (O.D.) at 600 nm reached 1.0. The cells were then washed with a buffer consisting of 97% 1× PBS and 3% LB and diluted to an O.D. 600 of 0.4 in the same buffer. A mixture of 50 μL of serially diluted peptide and 20 μL of dye (ONPG, final concentration 3 mM) was added to 100 μL of the bacterial suspension in a sterilized 96-well plate. Melittin served as a positive control, while buforin-2 was used as a negative control. The hydrolysis of ONPG was measured using a PHOMO Elisa reader (Autobio, Zhengzhou, China) at 405 nm for each time period.

### 2.9. DNA-Binding Assay

We evaluated the DNA-binding ability of F2 and F2 (C4S) monomeric peptides using gel retardation experiments. The concentration of plasmid DNA (pHIS2) was fixed at 145 μg/mL. The peptide was serially diluted in a binding buffer consisting of 10 mM Tris-HCl (pH 8.0), 5% glucose, 50 μg/mL BSA, 1 mM EDTA, and 20 mM KCl. This solution was then mixed with the DNA and incubated at 37 °C for 1 h. After incubation, the DNA–peptide mixture was stained with DNA gel loading dye. The samples were analyzed using 1% agarose gel electrophoresis in 0.5× TAE buffer. Plasmid bands were detected with a UV illuminator (Bio-Rad, Hercules, CA, USA).

### 2.10. Hemolysis Assay

Sheep red blood cells (RBCs) were used to assess the hemolytic activity of F2. Fresh blood was gently washed with 1× PBS and centrifuged for 5 min at 3000 rpm to obtain purified RBCs, which were then resuspended in 1× PBS to achieve a 4% RBC solution. Each well of a sterilized 96-well plate received 100 μL of the serially diluted peptide and 4% RBCs, and the mixture was incubated at 37 °C for 1 h at 60 rpm. Subsequently, the plates were centrifuged at 1200 rpm for 5 min, and the supernatant was transferred to a new 96-well plate. The absorbance of the transferred supernatant was measured at 405 nm using a PHOMO Elisa reader (Autobio, China). The hemolytic activity was calculated as the percentage of hemolysis, determined by subtracting the absorbance of the negative control (containing only PBS buffer) from the absorbance of the peptide-treated samples. This value was then divided by the result obtained by subtracting the absorbance of the negative control from the absorbance of a 1% Triton X-100 solution, which served as a positive control indicating 100% hemolysis. The final value was multiplied by 100 to express the hemolytic activity as a percentage.

### 2.11. MTT Assay

An MTT assay was performed to evaluate cytotoxicity. Normal cells (Hs68) and cancer cells (HeLa) were each seeded in sterile 96-well plates using a 150 μL mixture of DMEM and 10% FBS and cultured for 24 h at 37 °C in a 5% CO_2_ atmosphere. Serial dilutions of the F2 dimer were administered to the cultured cells, which were then incubated for an additional 24 h. Subsequently, 20 μL of MTT solution (5 mg/mL in PBS buffer) was added to each well, and the plates were incubated for 4 h at 37 °C before the media were removed. The precipitated MTT formazan crystals were dissolved in 100 μL of DMSO for 5 min. Absorbance at 550 nm was measured using a PHOMO Elisa reader (Autobio, China). Cell viability was calculated by dividing the absorbance value (A550) of cells treated with the peptide by that of cells treated with buffer only, and the result was expressed as a percentage.

## 3. Results

### 3.1. Design of Rattusin Fragments

**Figure 1:**
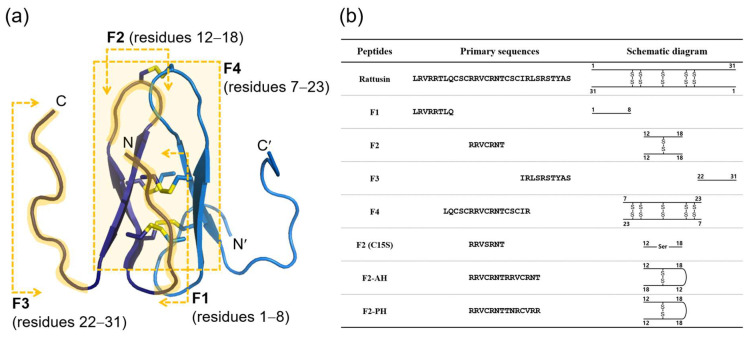
The design and sequences of rattusin fragments based on its three-dimensional structure. (**a**) The identification of fragment regions in the rattusin structure, with F1 (residues 1–8), F2 (residues 12–18), F3 (residues 22–31), and F4 (residues 7–23) highlighted in the full rattusin tertiary structure. The N-terminus (N) and C-terminus (C) are indicated for each fragment. (**b**) The primary sequences of the rattusin fragments alongside schematic diagrams representing the amino acid sequences and positions. Each fragment is depicted with its corresponding segment of the rattusin molecule, with disulfide bonds illustrated by lines connecting the cysteine residues.

Rattusin is characterized by its homodimeric structure, which is stabilized by five disulfide bonds, yet it exhibits relatively low antimicrobial activity considering the complexity of its structure. To pinpoint the functional region and identify the minimal peptide fragment derived from rattusin, we segmented the rattusin sequence into four distinct fragments: F1, F2, F3, and F4, as illustrated in Figure 1.

F1 corresponds to the N-terminal segment, spanning residues 1 to 8 (8-mer). F2 encompasses the dimeric hairpin loop region, which includes residues 12 to 18 and features a cysteine at position 15 (7-mer). F3 comprises the C-terminal segment, covering residues 22 to 31 (10-mer). F4 represents the dimeric core region, which is refolded by five disulfide bonds and comprises residues 7 to 23 (17-mer). Additionally, we synthesized the F2 (C15S) peptide, which was designed to prevent dimerization by substituting the cysteine at position 15. Furthermore, to elucidate the impact of F2 dimerization on its activity, we designed analogs of the F2 dimer: the antiparallel hairpin-like F2 (F2-AH) and the parallel hairpin-like F2 (F2-PH).

### 3.2. Preparation of Rattusin Fragments

Peptide fragments derived from the dimeric structure of rattusin were synthesized using solid-phase peptide synthesis (SPPS). Following synthesis, they were purified by RP-HPLC and characterized by LC-MS. Fragments F2, F4, F2-AH, and F2-PH, which contain either intra- or inter-disulfide bonds, underwent air oxidative refolding processes to achieve their oxidized forms. The purity of these peptides was confirmed to be greater than 95%, as evidenced by HPLC and LC-MS analyses (Table S1 and Figure S1).

### 3.3. Antimicrobial Activity of Fragments

The antimicrobial activities of the four fragments (F1, F2, F3, and F4) were initially evaluated against *E. coli* and *S. aureus*, with comparisons made to the activity of rattusin (Table 1). The MIC values obtained were consistent across all replicates, showing no deviations. The minimum inhibitory concentration (MIC) for rattusin was established at 58 μg/mL. Notably, F3 exhibited no antimicrobial activity at concentrations up to 590 μg/mL.

In contrast, F1 and F4 demonstrated MIC values that were either comparable to or slightly lower than that of rattusin. Remarkably, F2 displayed an MIC of 15 μg/mL, indicating antimicrobial activity fourfold greater than that of rattusin.

To elucidate the detailed structure–activity relationships of F2, we designed, synthesized, and tested various F2 analogs for their antimicrobial activity against nine different bacterial strains, including MRSA (Table 2). Among these, the F2 (C15S) variant, unable to form a dimer due to the absence of a cysteine residue, showed no antibacterial activity up to an MIC of 227 μg/mL. This observation underscores the critical role of dimer formation in the antimicrobial efficacy of F2. Furthermore, we developed the F2-AH and F2-PH analogs, characterized by continuously connected F2 sequences. Both analogs exhibited MIC values of 15 μg/mL, equivalent to that of the F2.

### 3.4. Membrane Depolarization

The examination of peptide-induced membrane permeabilization in intact *S. aureus* cells utilized the membrane-potential-sensitive dye DiSC(3)-5. This dye accumulates in the cytoplasmic membrane under normal membrane potential conditions, leading to fluorescence self-quenching. Membrane potential disruption causes the dye to disperse into the surrounding buffer, resulting in an elevated fluorescence intensity. The antimicrobial peptide melittin, known for membrane disruption, served as a positive control, whereas buforin-2, which does not interact with the membrane, functioned as a negative control.

**Figure 2:**
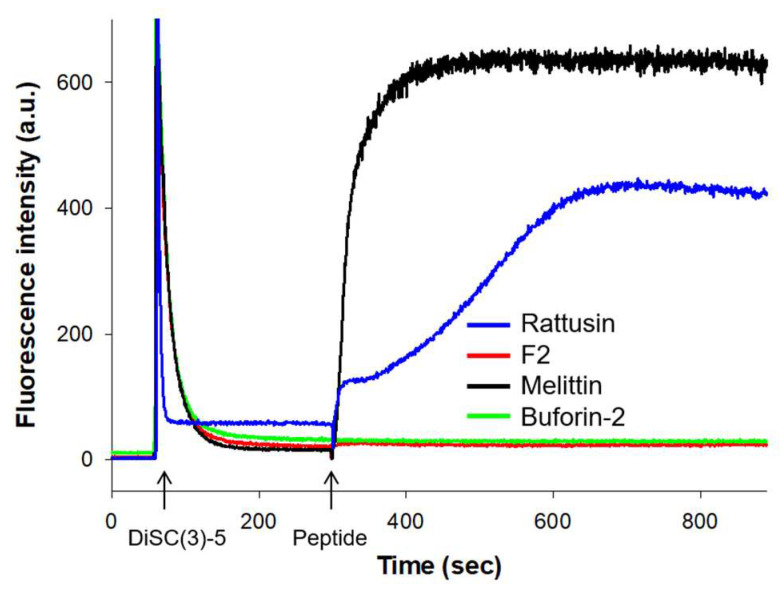
The time course of membrane depolarization in *S. aureus* induced by the antimicrobial peptide rattusin, its derivative fragment F2, and the control peptides melittin and buforin-2. Depolarization was monitored using the fluorescent DiSC(3)-5 dye. The arrows indicate the points of dye and peptide addition.

The initial strong quenching of DiSC(3)-5 fluorescence indicated the dye’s accumulation within the membrane. Following a stabilization period of 300 s, peptides were introduced (as indicated by an arrow in Figure 2), introducing a variable to the experiment.

Rattusin triggered a time-dependent depolarization of the bacterial cytoplasmic membrane, evidenced by a progressive increase in fluorescence intensity, attributable to the collapse of ion gradients that maintain the membrane potential. Melittin rapidly achieved the complete depolarization of *S. aureus* within seconds at a concentration of 4 μM. Conversely, buforin-2 failed to induce any change in membrane potential. Likewise, F2 did not cause any membrane depolarization, implying a distinctive mode of action on the bacterial membrane when compared to the rattusin peptide.

### 3.5. SYTOX Green Uptake

**Figure 3:**
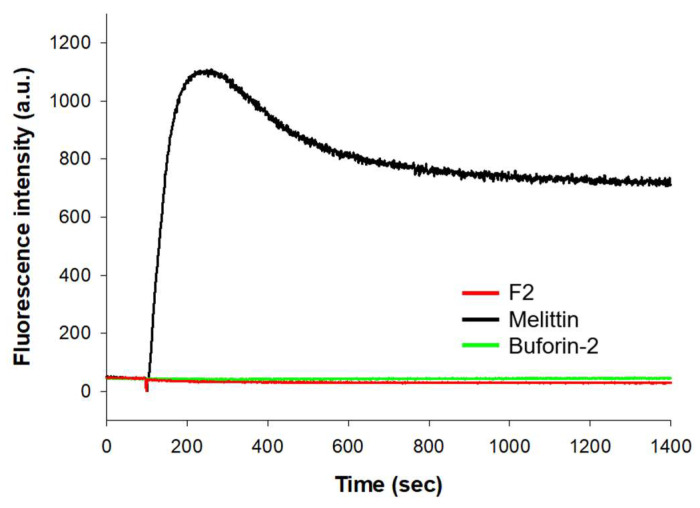
SYTOX Green uptake assay monitoring the permeabilization of *S. aureus* cell membranes by peptides. The concentrations of the peptides used in the experiment were 32 µM for F2, 4 µM for melittin, and 16 µM for buforin-2.

In the SYTOX Green uptake assay, which serves as an indicator of plasma membrane integrity, melittin demonstrated a significant increase in fluorescence intensity, indicative of its potent membrane-disruptive properties. This effect was rapid and pronounced, with melittin reaching peak fluorescence shortly after treatment application, as seen in Figure 3. Such a response is consistent with melittin’s established role in compromising membrane integrity, thereby allowing SYTOX Green to bind to nucleic acids and fluoresce. In stark contrast, the F2 fragment and buforin-2 did not show a similar increase in fluorescence intensity over the course of the assay. The absence of fluorescence enhancement with these peptides suggests that, unlike melittin, they do not cause membrane permeabilization under the conditions tested. Consequently, the data illustrate a clear distinction in the ability of melittin to disrupt cell membranes when compared to F2 and buforin-2.

### 3.6. ONPG Hydrolysis

**Figure 4:**
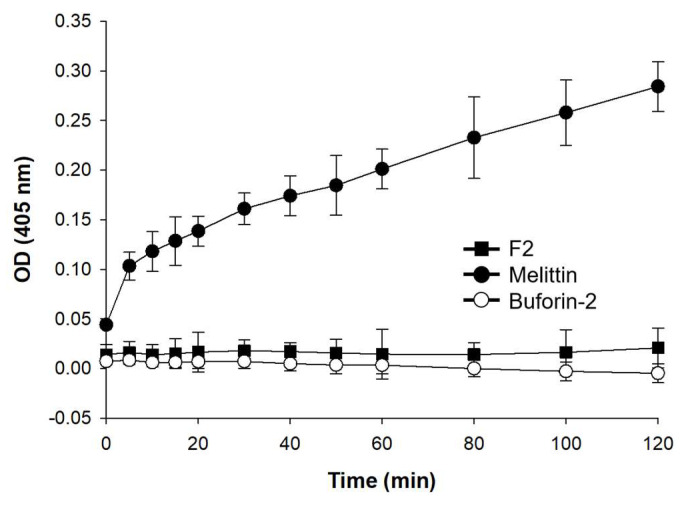
Assay of ONPG hydrolysis by peptides using *E. coli* ML-35. The concentrations of the peptides used in the experiment were 32 µM for F2, 4 µM for melittin, and 16 µM for buforin-2.

F2 appears not to interact with the membrane, as evidenced by depolarization and SYTOX experiments. We further investigated whether F2 could affect the inner membrane using an ONPG hydrolysis assay (Figure 4). For this purpose, *E. coli* ML-35 bacteria, from which the outer membrane had been removed, were utilized. Both ONPG and peptides were administered, and the absorbance was monitored at 405 nm. Melittin, known for its membrane-disrupting capabilities, served as the positive control, whereas buforin-2, which can traverse the membrane without causing destruction, acted as the negative control.

Despite the change in absorbance demonstrated by the positive control melittin, F2 did not alter the absorbance, similar to the negative control, buforin-2. This outcome, alongside the results from depolarization and SYTOX experiments, suggests that F2 does not target the inner membrane.

### 3.7. DNA-Binding Activity

The gel retardation assay elucidated the DNA-binding affinities of peptide variants. As hypothesized, F2 does not target the bacterial membrane, yet it manifests an augmented antimicrobial effect when contrasted with the wild-type rattusin, indicating an alternative mechanism of action. This is likely oriented toward the intracellular milieu, such as interactions with nucleic acids or proteins. To ascertain this, the assay was performed by mixing varying concentrations of the peptide with plasmid DNA.

**Figure 5:**
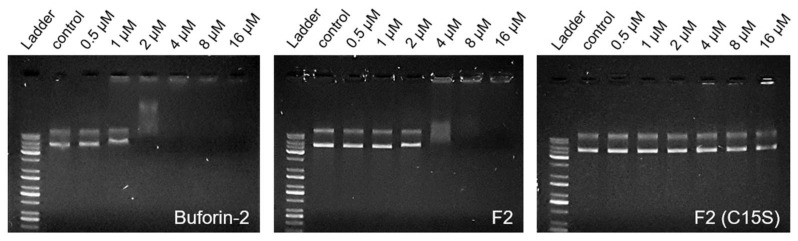
The DNA-binding activity of peptides assessed by the gel retardation assay. Gel electrophoresis illustrating the DNA-binding capability of peptides. The retarded bands signify successful DNA binding, observable for buforin-2 at 2 μM and F2 at 4 μM. No retardation was evident for the monomeric F2 (C15S) up to 16 μM.

Buforin-2, with established DNA-binding properties, was employed as a benchmark. At a concentration of 2 μM, buforin-2 successfully demonstrated DNA binding, evidenced by the retardation of the DNA band (Figure 5, left panel). This contrasts with F2, which required a doubled concentration of 4 μM to exhibit similar DNA retardation (Figure 5, middle panel). It is noteworthy that the monomeric variant of F2, specifically the C15S mutant, did not present any DNA retardation up to a concentration of 16 μM (Figure 5, right panel). We analyzed the DNA-binding images using ImageJ (1.54i) to quantify the DNA-binding activity of the peptides (Table S2). This lack of interaction suggests a pivotal role of dimerization in the DNA-binding process of F2.

### 3.8. Cytotoxicity

**Figure 6:**
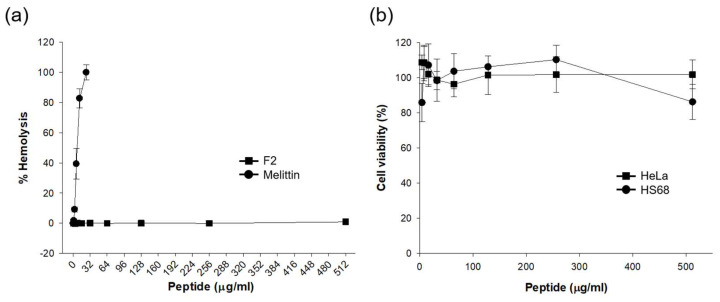
Cytotoxicity analysis of peptide F2. (**a**) Hemolysis assay results displaying the percentage of hemolysis of sheep RBCs after treatment with increasing concentrations of peptide F2 and melittin. (**b**) MTT assay results showing cell viability percentages for Hs68 and HeLa cells upon treatment with varying concentrations of peptide F2.

Cytotoxicity assays were conducted using hemolysis and MTT assays for F2, as depicted in Figure 6. In the hemolysis assay, sheep red blood cells (RBCs) were utilized, and F2 was serially diluted from 1 to 512 μg/mL. F2 did not induce hemolysis, even at concentrations up to 512 μg/mL, whereas melittin demonstrated 100% hemolytic activity at concentrations below 10 μg/mL. For the MTT assay, Hs68 cells and HeLa cells were employed. F2 exhibited no cytotoxicity at concentrations up to 512 μg/mL. These results indicate that F2 is highly specific to bacterial cells without affecting mammalian cells.

Further, the MTT assay was implemented to determine the effect of F2 on the viability of mammalian cells. Two cell lines, Hs68 (human fibroblasts) and HeLa (human cervical cancer cells), were treated with F2. The peptide maintained cell viability without notable toxicity up to 512 μg/mL for both cell types (Figure 6b). These findings delineate the specificity of F2’s antimicrobial action, highlighting its negligible cytotoxicity to mammalian cells and supporting its potential as a selective antimicrobial agent.

## 4. Discussion

Finding and improving the functional domains of current antimicrobial peptides (AMPs) is a potential strategy for creating new antibiotics. AMPs like rattusin, known for their antibacterial activities, often have complex structures and high molecular weights, which complicate their synthesis and clinical applications. This study focuses on rattusin, a C2 homodimeric peptide (2 × 33 amino acids) characterized by a robust range of antibacterial activities and significant salt resistance. However, the large size and complexity of rattusin make its synthesis challenging, hence the drive to design shorter, more manageable fragments that retain biological functionality.

We successfully designed and synthesized several short rattusin fragments (F1, F2, F3, and F4), targeting the functional site of the peptide based on its tertiary structure determined through NMR spectroscopy. These fragments were synthesized to explore the peptide’s active sequences and investigate the impacts of length and complexity on synthesis yield and antimicrobial activity.

Among the synthesized fragments, F2 exhibited superior antimicrobial properties, significantly outperforming the wild-type peptide. This fragment includes a central cysteine crucial for forming a disulfide-bonded dimer, key to its biological activity. The antimicrobial efficacy of F2 was notably abolished when the cysteine was replaced with serine (F2 (C15S)), highlighting the importance of the dimeric structure facilitated by disulfide bonding.

**Figure 7:**
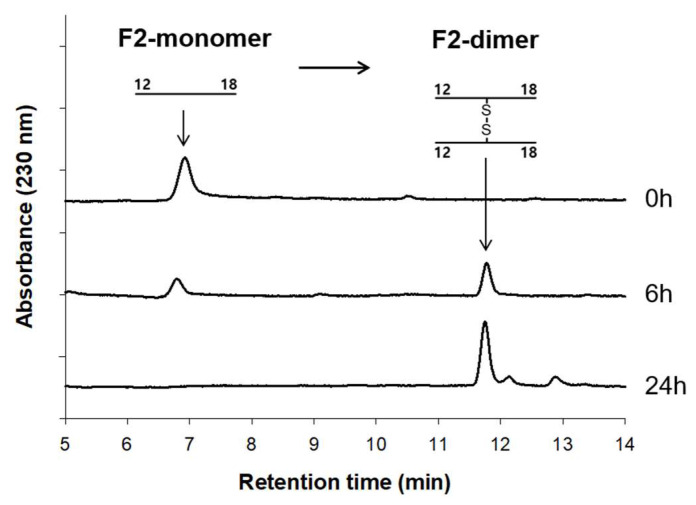
The dimerization of the F2 fragment. The formation of dimers of the F2 peptide monitored by RP-HPLC.

Interestingly, the monomeric form of F2 (prior to disulfide bonding) showed antimicrobial activity similar to that of the dimeric form. This observation suggests that the monomeric F2 can spontaneously form dimers under physiological conditions, which we confirmed using HPLC analysis after incubation in MIC buffer (Figure 7). This ability to dimerize in situ emphasizes F2’s therapeutic potential, allowing for simpler administration and potentially lower manufacturing costs.

A distinctive feature of F2, compared to the parent rattusin peptide, is its mechanism of action. Unlike rattusin, which disrupts bacterial membranes, F2 targets bacterial DNA directly. This mechanism was elucidated through fluorescence assays and DNA retardation experiments, which demonstrated that the F2 dimer binds to DNA effectively, inhibiting bacterial function at a fundamental level. This mode of action is particularly advantageous, as it reduces the likelihood of bacteria developing resistance, a common drawback of traditional antibiotics, which typically target membrane integrity or protein synthesis.

Moreover, the F2 dimer exhibited remarkably low cytotoxicity toward mammalian cells, as evidenced by hemolysis and MTT assays. It showed negligible hemolytic activity and did not affect cell viability at concentrations significantly higher than its MIC against bacteria. This selective toxicity, combined with its potent antimicrobial activity, positions the F2 dimer as an excellent candidate for further development into a new class of peptide-based antibiotics.

## 5. Conclusions

The findings from this study underscore the potential of designing shorter AMPs that not only retain but enhance the desirable properties of naturally occurring peptides. The F2 fragment, in particular, emerges as a compelling lead compound for new antibiotic development due to its effective DNA-binding capability and low mammalian cytotoxicity. These characteristics suggest that F2 and peptides like it could serve as foundational structures for the synthesis of new drugs aimed at combating antibiotic-resistant bacteria through mechanisms that differ fundamentally from those of current antibiotics. Future work will focus on optimizing the synthesis of F2 and similar peptides, expanding the range of bacteria against which these peptides are effective, and further delineating their safety profiles in clinical settings. The goal is to develop a new class of AMP-based antibiotics that leverage the unique properties of peptides like F2—targeting bacterial DNA and exhibiting minimal side effects—to provide a potent, safe, and economically viable alternative to traditional antibiotics.

## Figures and Tables

**Table 1:** Comparative minimal inhibitory concentrations (MICs) of rattusin and derived fragments against *E. coli* and *S. aureus* (μg/mL).

Bacteria	Rattusin	F1	F2	F3	F4
*Escherichia coli*	58	67	15	>590	32
*Staphylococcus aureus*	117	>500	58	>590	64

**Table 2:** MICs of F2 derivative peptides against Gram-negative and Gram-positive bacteria, including methicillin-resistant *S. aureus* (MRSA) (μg/mL).

Bacteria	F2	F2 (C15S)	F2-AH	F2-PH
**Gram-negative**				
*Escherichia coli*	15	>227	15	15
*Salmonella typhimurium*	15	>227	15	15
*Pseudomonas aeruginosa*	15	>227	15	15
**Gram-positive**				
*Staphylococcus aureus*	58	>227	29	29
*Bacillus subtilis*	29	>227	29	29
*Staphylococcus epidermidis*	15	>227	15	15
**MRSA**				
CCARM 3089	58	>114	29	29
CCARM 3090	116	>114	29	58
CCARM 3095	58	>114	58	29

## Data Availability

All relevant data are within the paper and Supplementary Materials.

## Supplementary Materials

The following supporting information can be downloaded at https://www.mdpi.com/article/10.3390/biom14060659/s1: Figure S1: Analysis of synthesized peptides. The purities and molecular masses of fragment peptides were confirmed using LC-MS. (a) F1, (b) F2, (c) F3, (d) F4, (e) F2 (C15S), (f) F2-AH, and (g) F2-PH. Table S1: Molecular masses of rattusin fragments and its analogs (theoretical and experimental data); Table S2: Quantitative analysis of DNA-binding activity of peptides. Mean values of DNA gel band intensities from the gel retardation assay (Figure 5), assessed using ImageJ (1.54i) software.
