# Supplementary material for: A Novel Dimeric Short Peptide Derived from α-Defensin-Related Rattusin with Improved Antimicrobial and DNA-Binding Activities
Source: Biomolecules. 2024 Jun 5;14(6):659. doi: 10.3390/biom14060659 (PMC11201828; doi:10.3390/biom14060659)
Supplement: Supplementary file 1 [file biomolecules-14-00659-s001.zip › biomolecules-3013375-supplementary.pdf]

Supplementary Materials

A novel dimeric short peptide derived from α-defensin related rattusin with improved antimicrobial and DNA binding activities

Gwansik Park[1,†], Hyosuk Yun[1,†], Hye Jung Min[2,*], and Chul Won Lee[1,*]

Table S1. Molecular masses of rattusin fragments and its analogs (theoretical and experimental data)

**Peptides**	**Molecular weight**						
	Calculated m/z				Observed m/z		
	MW	$[M+H]^+$	$[M+2H]^{2+}$	$[M+3H]^{3+}$	$[M+H]^+$	$[M+2H]^{2+}$	$[M+3H]^{3+}$
F1	1040.2	1041.2	521.1	347.7	1041.0	521.1	347.5
F2	1806	1807	904.0	603	-	903.7	602.5
F3	1152.3	1153.3	577.6	385.1	1152.9	577.3	577.2
F4	3993.8	3994.8	1997.9	1331.9	-	-	1331.1
F2 (C15S)	887	888	444.5	296.7	887	444.2	296.9
F2-AH	1787.1	1788.1	894.6	596.7	1787.3	894.7	597.2
F2-PH	1787.1	1788.1	894.6	596.7	1787.1	894.3	596.8

Table S2. Quantitative analysis of DNA binding activity of peptides. Mean values of DNA gel band intensities from the gel retardation assay (Figure 5), assessed using ImageJ software.

Peptides	Concentration (μM)						
	Control	0.5	1	2	4	8	16
Buforin-2	106.8	103.7	88.9	36.4	30.0	26.2	19.7
F2	103.2	95.1	83.6	96.0	42.0	24.7	18.2
F2 (C15S)	94.9	102.6	95.9	101.0	102.1	82.8	73.7

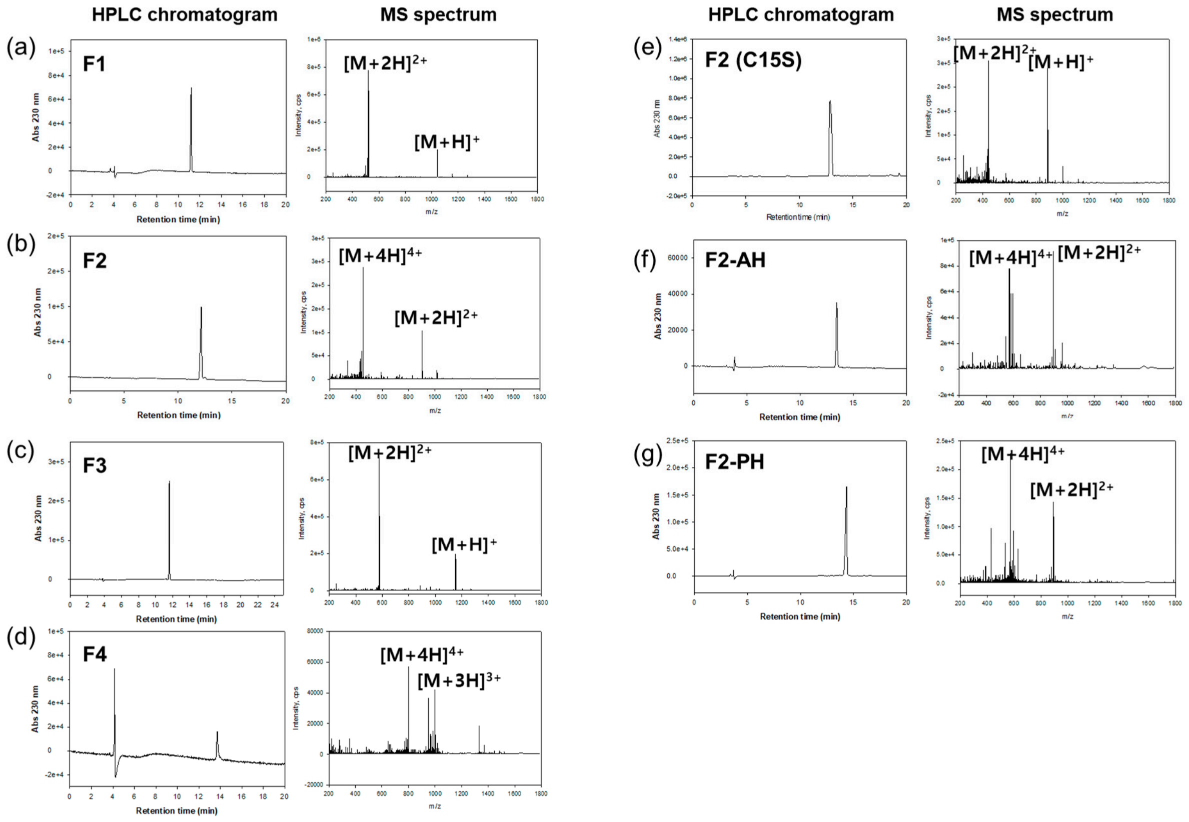

Figure S1. Analysis of synthesized peptides. The purities and molecular masses of fragment peptides were confirmed using LC-MS. (a) F1, (b) F2, (c) F3, (d) F4, (e) F2 (C15S), (f) F2-AH, and (g) F2-PH.
